# Home-Based Adaptation to Night-Time Non-Invasive Ventilation in Patients with Amyotrophic Lateral Sclerosis: A Randomized Controlled Trial

**DOI:** 10.3390/jcm11113178

**Published:** 2022-06-02

**Authors:** Eleonora Volpato, Michele Vitacca, Luciana Ptacinsky, Agata Lax, Salvatore D’Ascenzo, Enrica Bertella, Mara Paneroni, Silvia Grilli, Paolo Banfi

**Affiliations:** 1 IRCCS Fondazione Don Carlo Gnocchi, 20148 Milan, Italy; lptacinsky@gmail.com (L.P.); alax@dongnocchi.it (A.L.); dascenzo.salvatore@gmail.com (S.D.); s.silvia.grilli@gmail.com (S.G.); pabanfi@dongnocchi.it (P.B.); 2 Department of Psychology, Università Cattolica del Sacro Cuore, 20123 Milan, Italy; 3 Istituti Clinici Scientifici Maugeri IRCCS, Respiratory Rehabilitation of the Institute of Lumezzane, 25065 Brescia, Italy; michele.vitacca@icsmaugeri.it (M.V.); enrica.bertella@fsm.it (E.B.); mara.paneroni@icsmaugeri.it (M.P.)

**Keywords:** amyotrophic lateral sclerosis, ALS, motor neuron disease, MND, non-invasive ventilation, NIV, homecare

## Abstract

Background: Initiation to Non-Invasive Ventilation (NIV) in amyotrophic lateral sclerosis (ALS) can be implemented in an inpatient or outpatient setting. Aims: We aimed to evaluate the efficacy of adaptation (the number of needed sessions) to home-based NIV compared to an outpatient one in ALS in terms of arterial carbon dioxide (PaCO2) improvement. NIV acceptance (mean use of ≥5 h NIV per night for three consecutive nights during the adaptation trial), adherence (night-time NIV usage for ≥150 h/month), quality of life (QoL), and caregiver burden were secondary outcomes. Methods: A total of 66 ALS patients with indications for NIV were involved in this randomized controlled trial (RCT): 34 underwent NIV initiation at home (home adaptation, HA) and 32 at multiple outpatient visits (outpatient adaptation, OA). Respiratory function tests were performed at baseline (the time of starting the NIV, T0) together with blood gas analysis, which was repeated at the end of adaptation (T1) and 2 (T2) and 6 (T3) months after T1. NIV adherence was measured at T2 and T3. Overnight cardiorespiratory polygraphy, Short Form Health Survey (SF-36), Caregiver Burden Inventory (CBI), Caregiver Burden Scale (CBS), and Zarit Burden Interview (ZBI) were performed at T0, T2, and T3. Results: Fifty-eight participants completed the study. No differences were found between groups in PaCO2 at T1 (*p* = 0.46), T2 (*p* = 0.50), and T3 (*p* = 0.34) in acceptance (*p* = 0.55) and adherence to NIV at T2 and T3 (*p* = 0.60 and *p* = 0.75, respectively). At T2, the patients’ QoL, assessed with SF-36, was significantly better in HA than in OA (*p* = 0.01), but this improvement was not maintained until T3 (*p* = 0.17). Conclusions: In ALS, adaptation to NIV in the patient’s home is as effective as that performed in an outpatient setting regarding PaCO2, acceptance, and adherence, which emphasizes the need for further studies to understand the role of the environment concerning NIV adherence.

## 1. Introduction

The most common cause of death in amyotrophic lateral sclerosis (ALS) is respiratory failure due to atrophy and weakness of the respiratory muscles. Diaphragmatic dysfunction can be the first manifestation, or it can develop later as the disease progresses [1]. The use of non-invasive ventilation (NIV) has markedly increased during the last two decades and is now an integral part of the management of both acute and chronic respiratory problems in different clinical conditions [2]. Since 2015, in Italy, home-based adaptation to NIV has become an integral part of the care options for ALS patients, re-serving the inpatient and outpatient settings for patients who are experiencing an acute decline or who require multiple therapies, multidisciplinary diagnosis, or need close nocturnal observation. Recent technological advances and the increased capability to remotely monitor ventilation have facilitated the use of the home’s adaptation to NIV, where team and skills experiences are relevant. It has been shown that patient compliance to NIV can slow pulmonary function decline in ALS [3], avoid or reduce the need for hospitalization, improve quality of life (QoL), and lengthen survival [4]. One study has recently shown that very early NIV initiation can improve survival in ALS patients [5]. A study by Bertella et al. showed that outpatient NIV initiation is not inferior to inpatient NIV initiation in ALS in terms of patients’ acceptance and adherence [6,7,8,9]. Indeed, Sheers and colleagues [6] have shown that outpatient adaptation to NIV may also be the most appropriate solution.

Studies on home use of NIV have mostly focused on usage but not on the home as a setting for training and adaptation to NIV. Furthermore, the results have not always been noteworthy, probably due to poor initial monitoring opportunities or because the studies were exclusively dealing with NIV modalities [10]. Unfortunately, home-based NIV adaptation in ALS patients has not been sufficiently assessed as an option in standard care. It has not been established what the best setting for NIV adaptation is (i.e., a hospital, outpatient clinic, or home, with or without telemonitoring) [6,7,11,12]. To our knowledge, there are no previous studies that demonstrated which is the best setting for NIV adaptation (i.e., outpatient clinic, hospital, home, or telemonitoring) [6,7,11,12]. Currently, there is a tendency to avoid hospitalization by promoting a different and less stressful approach for ALS patients. Nevertheless, it is possible to detect differences across countries: while patient hospitalization to initiate NIV in the United States is uncommon [13], in Europe, Japan [14], or China [15], hospitalization remains the first choice [6,7,11].

### Objectives

The main aim of this study was to examine if home-based adaptation to NIV (the number of needed sessions) in ALS patients is effective compared to the outpatient setting in terms of arterial carbon dioxide (PaCO2) improvement. As secondary aims, we evaluated NIV acceptance (mean use of ≥5 h NIV per night for three consecutive nights during the adaptation trial) and adherence (nocturnal NIV usage for ≥150 h/month), patient and caregiver satisfaction (satisfaction with NIV started in the two different settings), QoL, and the caregivers’ perceived burden [16,17].

## 2. Materials and Methods

This study was carried out following the Helsinki Declaration. All participants provided their signed informed consent at study entry, which was approved by the Ethics Committee (on 15 April 2015); Comitato Etico della Sezione IRCCS Fondazione Don Carlo Gnocchi, Board Affiliation: Comitato Etico IRCCS Regione Lombardia). The registration ID at ClinicalTrials.gov is NCT02537132.

### 2.1. Trial Design and Participants

In this randomized controlled bicentric trial, we consecutively enrolled patients with ALS, diagnosed according to the revised El Escorial criteria [18], who were referred to the ALS outpatient clinics of the Heart–Respiratory Rehabilitation Unit of the IRCCS Fondazione Don Carlo Gnocchi, Milan (Italy) and the Respiratory Rehabilitation Unit of the Istituti Clinici Scientifici Maugeri IRCCS, Institute of Lumezzane, Brescia (Italy) between May 2015 and December 2017 for respiratory function assessment.

Inclusion criteria were age ≥ 18 years with a clinical indication for NIV according to EFNS criteria [2]. Exclusion criteria were refusal to participate in the study; the presence of severe cardiac/pulmonary comorbidity, as a contraindication to NIV; distance from hospital >40 km or other problems to reach the outpatient clinic; severe bulbar weakness (ALSFRS-R Bulbar score < 9), also ascertained by the first neurologist’s evaluation; and cognitive impairment that would preclude understanding the study protocol. This latter item was ascertained using the validated Italian version of the Edinburgh Cognitive and Behavioural ALS Screen (ECAS) [19,20]. For clarity, the total score cut-offs were 97 (age ≤60 years, low–middle education) and 89 (age > 60 years, low–middle education); 108 (age ≤60 years, high education) and 107 (age > 60 years, high education).

NIV was indicated according to the following criteria: the presence of hypoventilation symptoms (dyspnoea, orthopnoea, paradoxical respiration, daytime fatigue and hyper-somnolence, and morning headache), morning arterial carbon dioxide tension (PaCO2) > 45 mmHg, significant nocturnal desaturation measured by pulse oximetry (SpO2) < 90% at night (%sleepSpO2 < 90 or T90) being >5%, forced vital capacity <80% of predicted value, and Pi max < −60 cmH_2_O.

### 2.2. Measures

Data concerning age, sex, body mass index, spinal or bulbar onset, and time from ALS onset to NIV were collected.

**Figure 1:**
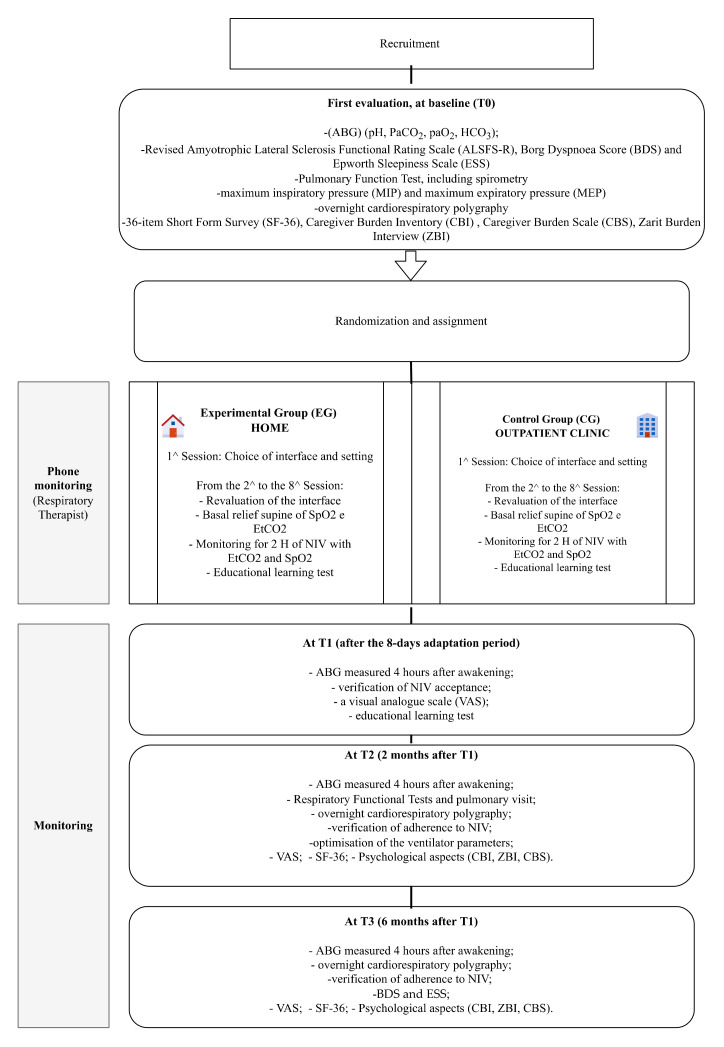
Scheme of the implementation procedure.

At baseline (the time of starting the NIV, T0), the patients underwent (Figure 1):

- Arterial blood gas analysis (ABG) (pH, PaCO2, PaO2, HCO3) measured 4 h after awakening;
- Clinical assessment, with the Revised Amyotrophic Lateral Sclerosis Functional Rating Scale (ALSFS-R) [21], Borg Dyspnoea Score (BDS) [22], and Epworth Sleepiness Scale (ESS) [23];
- Pulmonary function testing, including spirometry, performed following the European Respiratory Society guidelines [24], with the patient in a seated and supine position via a flanged mouthpiece, and using the suggested reference values [25], forced vital capacity (FVC), forced expiratory volume in the first second (FEV_1_) and FEV_1_/FVC% (Master Screen Body Jaeger Vyntus™ Pneumo, Vyaire, Mettawa, IL, USA);
- Maximum inspiratory pressure (MIP) and maximum expiratory pressure (MEP) (MicroRPM Pressure Meter, Micro Medical Ltd., Lewiston, ME, USA) via a flanged mouthpiece while the cheeks were held. Three measurements for a total of eight performed with less than 5% variability were recorded, and the highest value was used for the data analysis (patients with MEP < 60 cmH2O were provided with a Cough Assist device) [26,27];
- Overnight cardiorespiratory polygraphy with a nasal flow sensor, thoracic and abdominal effort measured with inductive plethysmography, and finger pulse oximeter (Embletta™ PDS, Medicare, Iceland), according to the American Academy of Sleep Medicine clinical practice guidelines: apnoea and hypopnea were scored manually using standard criteria [28,29];
- 36-item Short Form Survey (SF-36) [30], a questionnaire on the patient’s health status;
- The caregiver Burden Inventory (CBI) [31], Caregiver Burden Scale (CBS) [32], and Zarit Burden Interview (ZBI) [33] are designed to detect the practical and psychological burden of the caregiver. These tools were used both at baseline and at the end of the study (6-month follow-up).
- At T1 (after the 8-day adaptation period), the following evaluations were performed:
- ABG measured 4 h after awakening;
- Verification of NIV acceptance, i.e., mean use of ≥5 h NIV per night for 3 consecutive nights during the adaptation trial;
- A visual analog scale (VAS), on which the patient indicated the degree of satisfaction with NIV management and nursing assistance (0–10, low–high);
- Educational learning test designed to verify the knowledge and skills acquired by the patient concerning the path taken by the physiotherapist (see Supplementary Material Table S1).

At T2 (2 months after T1), the patients underwent:

- ABG measured 4 h after awakening;
- Overnight cardiorespiratory polygraphy with airway pressure proximal to the mask thoracic and abdominal effort measured with inductive plethysmography and finger pulse oximeter;
- Verification of adherence to NIV (night-time NIV usage for ≥150 h/month);
- Optimization of the ventilator parameters [34];
- VAS, SF-36, CBI, CBS, and ZBI (as above).

At T3 (6 months after T1), the following evaluations were performed:

- ABG measured 4 h after awakening;
- Overnight cardiorespiratory polygraphy;
- Verification of adherence to NIV;
- BDS and ESS;
- VAS, SF-36, CBI, CBS, and ZBI.

### 2.3. Intervention: The NIV Adaptation Trial

Participants were randomized to the following groups:

- *Home Adaptation* (*HA*)*:* in addition to the usual medical care, patients received no fewer than 8 sessions in the afternoon for about two hours at home with assistance provided by the Respiratory Therapist (RT) to help them adapt to NIV and education on the management of bronchial secretions.
- *Outpatient Adaptation* (*OA*)*:* in addition to the usual medical treatment, patients attended no fewer than 8 sessions in the afternoon for about two hours in the outpatient clinic to help them adapt to NIV and were educated about the management of bronchial secretions.

At the first access in both groups, the interface and respiratory settings were selected (Trilogy or BiPAP^®^ and AVAPS^®^, a specific device that offers a bi-level ventilation mode allowing for application of an average tidal volume, Philips Respironics, Murrysville, PA, USA) in spontaneous/timed mode or pressure-controlled mode with a pre-set tidal volume of 7 mL/Kg and a fixed respiratory backup rate of 12 breaths/minute [35,36]. At least a 2 h trial of NIV was conducted to monitor SpO2 and end-tidal carbon dioxide (ETCO2) using a CO2SMO monitor (Novametrix, Respironics, Carlsbad, CA, USA). It provides reliable mainstream measurement and display of ETCO2 and respiratory rate under direct RT supervision. During this period, if SpO2 < 94% or ETCO2 > 45 mmHg, RT increased IPAP or EPAP until values normalized. A non-vented facial mask was connected to the CO2SMO probe, and the latter was connected to a whisper swivel (Koninklijke Philips Electronics N.V., Amsterdam, The Netherlands). Patients were recommended to use NIV only during the night and as much as possible until they had completely adapted to NIV. During the adaptation period, educational sessions were provided to each patient to ensure that the NIV use was adequate and that the ventilator was being properly managed (max. 8 sessions/patient). After each session, patients expressed their level of satisfaction with VAS. No inspired gas conditioning system was used during adaptation to NIV.

#### 2.3.1. Criteria for a Correct Adaptation to NIV

The adaption to NIV was considered correct if the patient was able—with or without the caregiver’s help—to put on the interface, manage the ventilator and the alarms, and clean the ventilator components.

The data obtained from the 2 h monitoring were used to determine an average SpO2 > 94% and average ETCO2 < 45 mmHg in patients with daytime hypoventilation [37].

The educational learning test given at the end of each session had to be passed (see Supplementary Material Table S1). There was no objective difference between OA and HA in professional contact time and written information received. Our centers have a team of respiratory therapists who are specially trained in the NIV’s initiation.

#### 2.3.2. Criteria for Acceptance of NIV

Patients were considered adapted to NIV and they could interrupt the trial when: (1) they used NIV ≥ 5 h/night for 3 consecutive nights verified by the data collected from the ventilator software; (2) patients were able to accurately wear the mask, manage the ventilator and the alarms, and clean the ventilator components. On the other hand, the NIV adaptation was interrupted when (3) patients had failed to achieve NIV acceptance after 8 consecutive educational sessions, and (4) caregivers properly applied the mask to the patients.

#### 2.3.3. Sample Size

An initial power analysis was conducted using G*Power version 3.1.9.3 [38]. We defined a non-inferiority margin of 0.4 kPa (3.75 mmHg) for the difference in change of the primary endpoint, PaCO2, between home and outpatient initiation, as a difference less than 0.4 kPa was meant to be clinically irrelevant. This preliminary statistical analysis was performed considering previous studies with NIV’s PaCO2 changes of more than 0.45 kPa [39,40,41]. With a one-sided alpha of 0.05, a beta of 0.2, a Standard Deviation (SD) of 0.71, and an expected drop-out rate of 25%, at least 57.5 participants needed to be randomized.

#### 2.3.4. Randomization, Sequence Generation, and Allocation Concealment

Eligible patients were allocated to HA or OA (i.e., home-based vs. outpatient clinic-based NIV adaptation) using a method of minimization, considering baseline bulbar function, baseline FVC, age, and sex as the minimization factors.

A centralized, web-based randomization system was used to assign treatment allocation. A site-specific username and password were used to gain access to the system. Researchers were invited to enter patient details (identification number, date of birth, and the minimization factors) and to confirm consent and eligibility when completed. Then, the randomization system notified the user and the study manager of the treatment allocation.

### 2.4. Statistical Analysis

A complete statistical analysis plan was designed and approved before any analysis was carried out. Statistical analysis was performed using the Statistical Package for Social Science (SPSS, IBM^®^ version 24, IBM Corp., Armonk, NY, USA). Socio-demographic data and clinical information were expressed as mean ± Standard Deviation or as median and interquartile range. To compare the two groups and analyze interaction effects on outcome and process measures, analysis of variance (ANOVA) with two groups and three time periods was used. When there were significant interaction effects, *t*-tests were used to analyze the difference in pre- and post- groups. Similarly, between-group comparisons were performed using Welch’s test for unequal variances. H0 was rejected if the lower limit of the 95% confidence interval (CI) was less than the non-inferiority margin. Results were considered significant if *p* < 0.05.

## 3. Results

**Figure 2:**
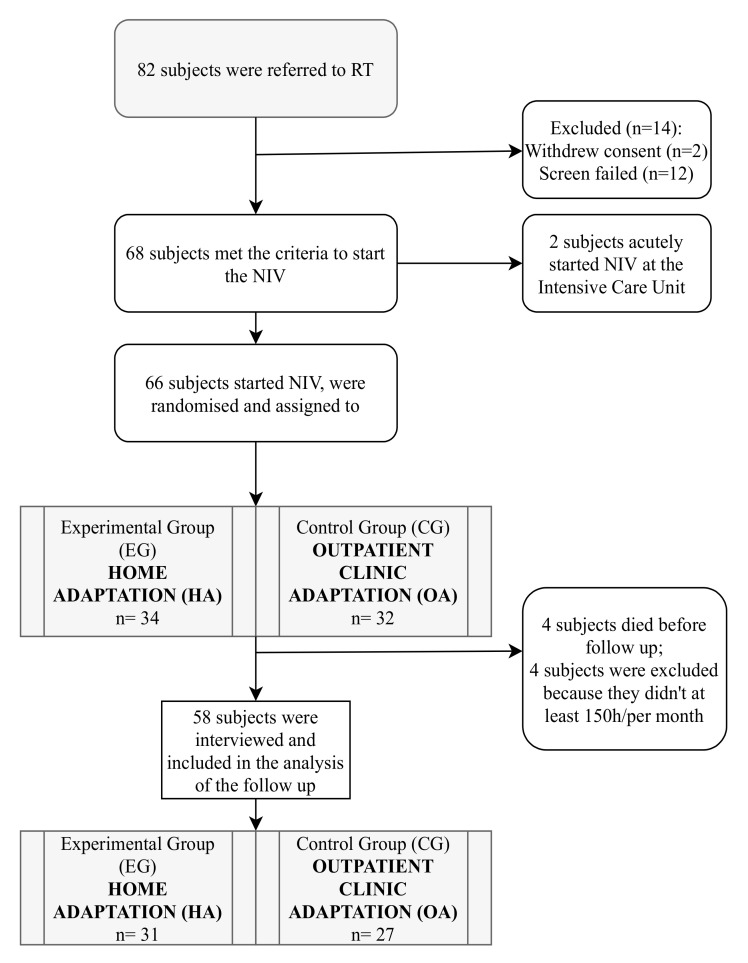
Flow chart of participant inclusion.

Out of 82 ALS patients referred for evaluation for NIV initiation during the study period, 68 met the criteria to start NIV. Of these, two patients were pre-emptively initiated on NIV in the Intensive Care Unit because of an episode of acute respiratory failure. Therefore, 66 subjects started NIV and were randomized to HA (n = 34) or OA (n = 32) (Figure 2).

The population was Italian-speaking, Caucasian, and mostly female (54.5%). The baseline characteristics are illustrated in Table 1.

A total of 58 participants completed the study. Overall, four participants (6.9%), two in OA (6%) and two in HA (5.9%), did not reach the goal of 150 h/month prescribed, and four participants died (6.9%), two in HA (5.9%) and two in OA (6%), and they were withdrawn from the study. Among the four participants who did not reach the 150 h/month target, two (OA) did not perceive the need for NIV and rejected it, one (HA) was a bulbar onset patient who had trouble using NIV for the sialorrhea problems, and one (HA) preferred to only use NIV during the day (Figure 1). No significant side effects were detected in the remaining ALS patients in the two study groups.

Table 2 shows that the baseline differences between groups in terms of the ABG analysis and overnight cardiorespiratory polygraphy did not change significantly at follow-up. In both groups, PaCO2 significantly improved during the 2-month follow-up but not during the 6-month follow-up.

**Figure 3:**
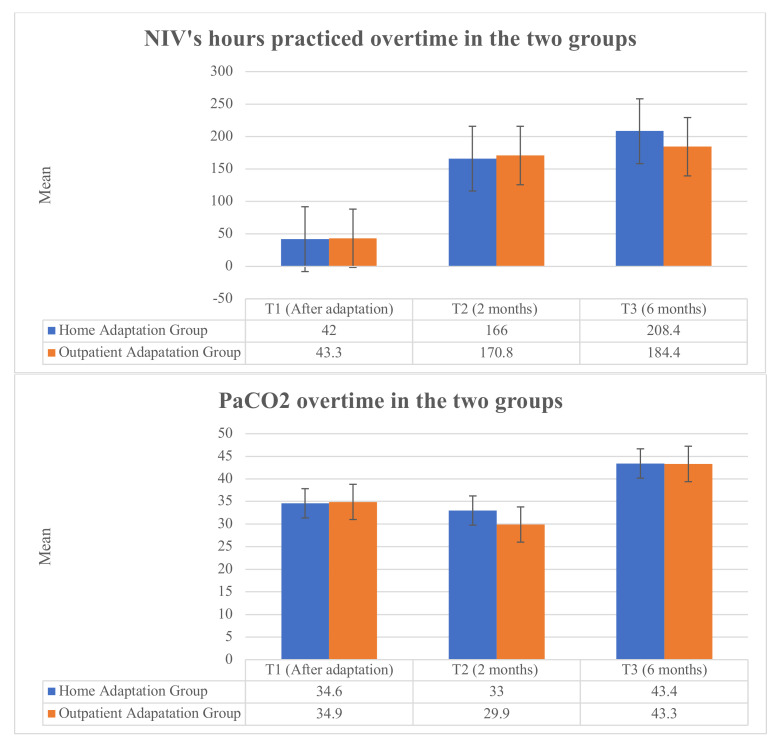
PaCO2 and NIV adherence (hours of NIV used/month).

Ten patients belonging to HA (29.4%) complained of difficulties in adapting to NIV due to: more than eight sessions required for the adaptation (three patients, 8.8%), pain or nose lesions (four patients, 11.8%), or low perceived need for NIV (three patients, 8.8%). These patients required two more sessions than the others to correctly adapt to NIV. On the other hand, 12 participants in OA (37.5%) also needed more than 8 sessions to adapt to NIV. Nevertheless, no significant differences in terms of NIV hours of usage were found between the groups (F (1, 42.3) = 0.27, *p* = 0.60) at T2 and (F (1, 40) = 0.10, *p* = 0.75) at the 6-month follow-up (Figure 3).

**Figure 4:**
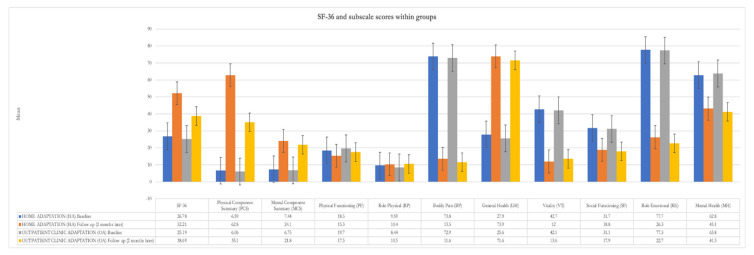
Development of scores from the SF-36 subscales before and after adaptation to NIV in the two settings.

At the 2-month follow-up, QoL, assessed with the SF-36, improved more significantly in HA (F (1, 55.82) = 6.98, *p* = 0.01) than in OA. This result, however, was not maintained over time at the 6-month follow-up (F (1, 1.94) = 42.3, *p* = 0.17). Moreover, only the General Health subscale of the SF-36 reported a significant improvement between the groups in favor of home adaptation at the 6-month follow-up (Table 3). Figure 4, on the other hand, shows the changes within the groups in terms of the subscales of the SF-36, in which there is a significant improvement in the Physical Component Summary (F (1, 62) = 68.04, *p* < 001) in both groups. However, despite an improvement in General Health, both groups showed a worsening in Bodily Pain (F (1, 62) = 3.59, *p* = 0.063), Role Emotional (F (1, 62) = 0.87, *p* = 0.355) and Mental Health (F (1, 62) = 0.41, *p* = 0.840) over time.

Considering the perceived burden of caregivers and its effect on their QoL, assessed with the CBS, no differences were found between the groups at either 2 (F (1, 25) = 1.29, *p* = 0.27) or 6 months (F (1, 25) = 0.00, *p* = 0.93). However, significant differences were found within HA between T1 and T2 (t (17) = 3.03, *p* = 0.00) but not within OA (t (18) = 2.08, *p* = 0.05) (Table 4).

Physical, psychological, and social burdens examined with both the CBI and ZBI only significantly improved immediately after the adaptation process to NIV in OA (CBI: *p* = 0.01; ZBI: *p* = 0.00) but not in HA (CBI: *p* = 0.18; ZBI: *p* = 0.05) (Table 3).

Finally, the overall degree of satisfaction with the adaptation to NIV, measured by VAS at the end of the NIV adaptation, was significantly higher in HA than OA (F (1, 26) = 7.48, *p* = 0.01). However, this result was not maintained at T2 (F (1, 59) = 0.35, *p* = 0.55) and T3 (F (1, 48) = 0.43, *p* = 0.51).

## 4. Discussion

In our study, the primary outcome was PaCO2 equality between home and outpatient NIV adaptation and its maintenance over time. PaCO2 initially increases during sleep, leading to nocturnal hypoventilation and, thus, diurnal hypercapnia. Hypercapnia leads to clinical symptoms such as headaches on awakening, daily fatigue, sleep disturbances, and depression. Starting NIV at home, in our study, was shown to be as effective in reducing CO2 as starting it in an outpatient setting.

ABG was performed at least 4 h after removing night-time ventilation to check whether the daytime hypoventilation state remained, demonstrating that there was no difference in PaCO2 values between the two groups over time. However, a significant difference (*p* = 0.02) was found between the PaCO2 measured at T2 compared with that at T3: this was due to the progression of the disease, although the patient increased the number of hours of NIV during the day. However, this increase in ventilation hours at 6 months did not maintain PaCO2 at similar levels compared with those measured at T2. Dorst [11] showed that ventilation times naturally increase due to clinical worsening to the point of 24 h of ventilation. Markovic [42] demonstrated that ALS patients, after 3 months of adaptation to NIV, increased their ventilator use hours, demonstrating the natural progression of respiratory dysfunction as the disease worsened. Manera et al. found that in 186 post-NIV ALS patients after NIV start, PaCO2 levels were not correlated to survival in ALS patients, and only HCO3 and Subacute Bacterial Endocarditis (SBE) levels were predictive of death or tracheostomy, and the risk for death/tracheostomy was increased by more than 40%, and survival was significantly shortened. Unfortunately, the limitation of this paper is that it does not describe how patients’ adherence to NIV was assessed [43]. Furthermore, the ABG data are often influenced by numerous factors such as the use of diuretics, vomiting, sodium retention, or ingestion of alkaline substances. In our study, three participants in the HA group and one participant in the OA group had a tracheostomy. Four participants died (6.9%), two in HA (5.9%) and two in OA (6%), and they were withdrawn from the study.

The evaluation of ETCO2, compared to transcutaneous CO2 (TcCO2), has been a debated issue for many years. In our work, a non-vented facial mask was connected to the CO2SMO probe, and the latter was connected to a whisper swivel to avoid leaks as much as possible: if SpO2 < 94% or ETCO2 > 45 mmHg, RT increased IPAP or EPAP until values were normalized. In this way, we checked for fixed leaks and then that the ETCO2 was stable throughout the afternoon monitoring period. We have also chosen this monitoring system because, compared to TcCO2, it is lighter and easily transportable at home. Sung-Min Kim [37], in his paper, demonstrated that brief waking supine capnography (EtCO2) could be useful as a screening tool for nocturnal hypoventilation and compliance with subsequent NIV treatment.

Perceptions of difficulties with NIV acceptance reported by patients were similar in the two groups. Our results are similar to those obtained by Chatwin et al. [8] in non-ALS neuromuscular patients, in whom outpatients increased their hours of night-time ventilation more than inpatients. In our study, the acceptance of NIV during home adaptation was the same as in the outpatient group. Our results show high feasibility within the home NIV adaptation group compared to the outpatient adaptation group that needed more than eight sessions in a higher percentage (29.4% in HA vs. 37.5% in OA). Moreover, ALS patients’ adherence to NIV at 2 and 6 months indicate that outpatient and home-based initiation of NIV are equivalent: only eight patients did not complete the study (four participants (6.9%), two in OA (6%), and two in HA (5.9%), did not reach the goal of 150 h/month prescribed, and four participants died (6.9%), two in HA (5.9%) and two in OA (6%)].

There are no Consensus Guidelines for NIV regarding an optimal monitoring strategy [44], adherence goals, or the best follow-up testing [44]. In a prospective controlled study of ALS patients using NIV at home, Pinto et al. [12] demonstrated that NIV adjustment was successfully managed through telemonitoring and that NIV compliance was comparable to that of evaluated outpatients. This study emphasizes how telemedicine improves survival and functional status in ALS patients and likely reduces disease costs. In our study, the home setting for NIV adaptation showed a greater improvement in the patients’ QoL but not in the caregiver’s burden. On the other hand, based on the results of the SF-36 administration, no significant differences emerged between the groups in terms of the different subscales, except for General Health, and some domains, such as Vitality or Social Functioning, showed stabilization or even deterioration compared to baseline, confirming the results of other studies that have explored QoL change in the past [45]. The physical, psychological, and social burdens of the respective caregivers only improved in the OA and not in the HA, suggesting the importance of the perceived safety for the person who is constantly at the patient’s side. In selecting which setting is most appropriate for NIV adaptation, it is important to consider factors such as the patient’s transportation availability, distance from the hospital, the presence of a competent caregiver at night, severe bulbar weakness, and any anxiety or cognitive issues the patient may have [46,47,48]. Patients who have the option of starting NIV at home are obviously in a more comfortable environment than in the hospital. Our home patients reported being in their environment with the help of family and being able to sleep as much as needed, whereas patients seen in an outpatient setting were in an impersonal environment without the usual landmarks, except for caregiver support.

Our data are in line with the systematic review by Macintyre et al. [49] and the study of Bourke et al. [50] that showed an improved time-weighted mean HRQoL in ALS patients treated with NIV. However, they are in contrast with previous studies showing that the QoL of ALS patients tends to worsen, compared to the non-ALS group, after 6 months of NIV [45]. On the other hand, as Hazenberg et al. noted in their observational study, methodological challenges regarding the measurement of QoL over time in ALS patients because of disease progression go hand-in-hand with a change in QoL [45].

The results of our study indicate that patients who prefer OA or HA initiation may be allowed to choose between the two. Factors to consider are the distance to the referral center, whether they have cognitive or bulbar difficulties, anxiety, and the presence of a caregiver who is knowledgeable and familiar with the patient’s needs.

### Limitations

An accurate cost analysis was not performed in this study, but we can speculate that home-based NIV initiation is cost-effective, considering that the Italian health system reimbursement for 1 day of hospitalization for an ALS patient in a rehabilitation center is EUR 370.37/day, and for one outpatient visit is EUR 230/day, while the reimbursement for home-based adaptation is EUR 47.00/day. The hourly cost of a physiotherapist at home, according to the regional health service, is EUR 23.50 per access. In our case, two accesses per day were reimbursed. However, the costs of NIV are high, limiting the ability to extend the time of adaptation, especially given the out-of-pocket costs that are likely to significantly increase the economic burden. Indeed, Meng and colleagues found that monthly costs tend to increase nine months before diagnosis, with a significant increase in the index month (Medicare: USD 10,398; commercial: USD 9354), which persists post-indult. In addition, prescriptions and equipment costs are burdensome in the post-diagnosis period, reaching 70.2% of the annual cost trend due to disease progression, with 9% of total costs associated with the disease (USD 126,161 over a 10-year disease duration) [51]. The limitations of our study also include the fact that it did not investigate in depth the subjective reality of adaptation to NIV in the two settings, an aspect that future research could investigate in greater depth using semi-structured interviews. The SF-36 sub-scales themselves, as well as emotional aspects such as anxiety or depression, could help in understanding the factors affecting the adaptation process to NIV, that, in other studies, are relevant in the adaptation process [46,47,48]. Finally, another limitation is that we only considered patients within 40 km, which is certainly different from the situation in many countries, where they may come from further distances.

## 5. Conclusions

In ALS, adaptation to NIV at the patient’s home is as effective as that performed in an outpatient setting in terms of improved PaCO2, acceptance, and adherence. QoL seems better when NIV is offered at home, whereas there seems to be no benefit in terms of caregiver burden. HA was preferred in stable patients and their caregivers and was probably less costly.

## Figures and Tables

**Table 1:** Baseline characteristics of the overall sample and both the experimental and the control group.

Characteristics	Overall Sample (n = 66)	Home Adaptation (HA) (n = 34)	Outpatient Adaptation (OA) (n = 32)	*p*-Value
**Female, n, %**	36 (54.5)	14 (41.2)	16 (50)	
**Age, y, mean (SD)**	69.1 (8.6)	67.4 (7.5)	70.9 (9.5)	0.11
**BMI, mean (SD)**	24.09 (6.7)	23.4 (2.9)	24.7 (4.3)	0.15
**Bulbar onset, n, %**	19 (28.8)	7 (20.6)	12 (37.5)	
**Spinal onset, n, %**	43 (65.1)	25 (73.5)	18 (56.2)	
**Respiratory onset, n, %**	4 (6.1)	2 (5.9)	2 (6.3)	
**ALS duration (from symptom onset), months, mean (SD)**	32 (4.7)	31.6 (5.2)	32.3 (4)	0.53
**FVC (% predicted)**	69.9 (23.8)	70 (23.09)	70 (21.4)	0.52
**FEV_1_ (% predicted)**	68 (24.3)	72 (22.8)	64 (25.8)	0.20
**FEV_1_/FVC (% predicted)**	99.3 (2.4)	98.8 (1.8)	99.7 (2.2)	0.40
**MIP (% predicted)**	40.2 (17.4)	44.7 (16.05)	35.2 (17.7)	0.26
**MEP (% predicted)**	47.2 (26.4)	37.9 (20.8)	43.3 (27.5)	0.25
**Ventilatory Mode, n, %**				
**S/T AVAPS**	30 (45.5)	13 (38.2)	17 (53.1)	
**S/T**	28 (42.2)	19 (55.8)	9 (26.5)	
**PACV**	7 (10.6)	2 (5.9)	5 (14.7)	
**APC/AVAPS**	1 (1.5)	0 (0)	1 (2.9)	
**Kind of masks used, n, %**				
**Respironics non-vented masks**	66 (100)	34 (100)	32 (100)	
**Comfort Gel Blue Full**	25 (37.9)	14 (41.2)	11 (34.4)	
**Easy Life**	18 (27.3)	9 (26.5)	9 (28.1)	
**Amara Gel**	23 (34.8)	11 (32.3)	12 (35.3)	
**ECAS, mean (SD)**	90.5 (6.82)	90.5 (3.8)	90 (9.03)	0.55
**ALSFRS-R, mean (SD)**	29.2 (6.6)	28.7 (6.4)	29.8 (6.9)	0.53
**ALSFRS-R Bulbar score, mean (SD)**	9.84 (1.04)	9.71 (1.11)	9.91 (0.99)	0.50
**BORG Dyspnoea Scale, mean (SD)**	0.49 (0.5)	0.39 (0.8)	0.59 (0.31)	0.19
**ESS, mean (SD)**	9.2 (0.4)	9.8 (0.3)	9.4 (0.4)	0.25

*Abbreviations:* SD = standard deviation; BMI = body mass index; ALS = amyotrophic lateral sclerosis; FVC = forced vital capacity; FEV_1_ = forced expiratory volume in the first second; MIP = maximum inspiratory pressure; MEP = maximum expiratory pressure; AVAPS = average volume-assured pressure support; PACV = pressure assist-control ventilation; ECAS = Edinburgh Cognitive and Behavioural ALS Screen; ALSFRS-R = Amyotrophic Lateral Sclerosis Functional Rating Scale-Revised; ESS = Epworth Sleepiness Scale; S/T = spontaneous time; APC = adaptive pressure control.

**Table 2:** Arterial blood gas analysis and polygraphy between groups.

Outcomes	Baseline			After Adaptation			Follow Up (2 Months Later)			Follow Up (6 Months Later)		
	HA n = 34	OA n = 32	95%Cl, *p* Value	HA n = 34	OA n = 32	95%Cl, *p* Value	HA n = 31	OA n = 27	95%Cl, *p* Value	HA n = 31	OA n = 27	95%Cl, *p*-Value
***ABG***												
**ph, mean (SD)**	7.39 (1.27)	7.42 (0.44)	7.32–7.42, *p* = 0.34	7.39 (2.8)	7.39 (3.1)	*p* = 0.34	7.39 (3.45)	7.41 (0.02)	7.35–7.37, *p* = 0.33	7.42 (0.02)	7.40 (14.6)	7.37–7.39, *p* = 0.33
**PaCO2, mean (SD)**	42.29 (5.56)	43.58 (8.17)	41.21–44.62, *p* = 0.46	34.6 (3.3)	34.9 (2.9)	*p* = 0.46	33.0 (17.24)	29.93 (19.16)	27.05–35.97, *p* = 0.50	43.4 (2.81)	43.3 (2.29)	38.0–48.0, *p* = 0.46
**PaO2, mean (SD)**	77 (9.13)	75.26 (9.84)	73.83–78.88, *p* = 0.46	78.1 (9)	77.4 (9.7)	*p* = 0.46	77.0 (5.79)	77.9 (6.71)	75.67–79.15, *p* = 0.61	78.3 (5.95)	76.27 (6)	68.0–91.0, *p* = 0.24
**HCO3, mean (SD)**	29.97 (3.95)	29.19 (3.72)	28.19–30.96, *p* = 0.80	27.8 (3.4)	28.1 (3.61)	*p* = 0.28	29.24 (2.16)	29.74 (1.74)	28.03–30.91, *p* = 0.37	29.95 (2.02)	31.04 (1.9)	24.2–33.0, *p* = 0.11
**SaO2, mean (SD)**	94.17 (2.17)	94.51 (2.8)	93.72–94.95, *p* = 0.59	95.2 (2.12)	94.9 (2.1)	*p* = 0.21	94.51 (1.47)	95.00 (1.38)	93.33–95.15, *p* = 0.24	94.86 (1.38)	94.53 (1.49)	92.0–97.0, *p* = 0.44
***Polygraphy***												
**AHI, mean (SD)**	13.34 (11.71)	19.99 (17.96)	12.58–20.22, *p* = 0.09	---	---	---	5.90 (4.99)	6.65 (5.83)	7.47–13.7, *p* = 0.09	5.20 (4.57)	7.11 (7.45)	4.58–7.66, *p* = 0.22
**SpO2, mean (SD)**	91.91 (2.13)	90.69 (3.51)	90.60–92.05, *p* = 0.10	---	---	---	91.8 (5.2)	92.1 (6.1)	89.1–93.5, *p* = 0.88	91.60 (16.25)	91.43 (16.76)	87.46–95.47, *p* = 0.96
**ODI, mean (SD)**	11.47 (10.36)	11.10 (7.52)	8.95–13.36, *p* = 0.76	---	---	---	8.6 (3.8)	8.15 (3.23)	1.72–2.44, *p* = 0.05	4.85 (4.91)	6.57 (8.20)	4.00–7.36, *p* = 0.31
**T90%, mean (SD)**	14.78 (26.03)	27.38 (34.78)	13.00–28.04 *p* = 0.10	---	---	---	7.61 (6.62)	5.7 (6.63)	2.34–2.54, *p* = 0.07	12.09 (6.07)	12.08 (2.06)	1.98–11.88, *p* = 0.14

*Abbreviations:* SD = standard deviation; HA = Hospital Adaptation; OA = Outpatient Adaptation; ABG = Arterial Blood Gas Analysis; ph = measurement of acidity or alkalinity; PaCO2 = partial pressure of carbon dioxide; PaO2 = partial pressure of oxygen; SaO2 = Oxygen Saturation (arterial blood); SpO2 = Spot peripheral capillary oxygen saturation; HCO3 = bicarbonate; AHI = Apnea Hypopnea Index; ODI = Oxygen Desaturation Index; T90% = total sleep time in oxygen saturation ≤ 90%. Data are reported in mmHg (1 kPa = 7.5 mmHg).

**Table 3:** Quality of life and caregiver burden result between groups.

	Baseline			Follow Up (2 Months Later)			Follow Up (6 Months Later)		
Outcomes	Home Adaptation (HA) n = 34	Outpatient Clinic Adaptation (OA) n = 32	95% Cl, *p*-Value	Home Adaptation (HA) n = 29	Outpatient Clinic Adaptation (OA) n = 23	95% Cl, *p*-Value	Home Adaptation (HA) n = 29	Outpatient Clinic Adaptation (OA) n = 23	95% Cl, *p*-Value
**SF-36, mean (SD)**	26.78 (7.92)	25.19 (9.71)	22.36–27.32, *p* = 0.150	52.21 (32.04)	38.69 (21.63)	41.43–56.66, *p* = 0.011	28.2 (3.57)	30.0 (4.80)	15.0–30.0; *p* = 0.171
***Physical Component Summary*** **(*PCS*)**	6.59 (3.88)	6.06 (3.92)	1.4–13.1, *p* = 0.636	62.8 (31.7)	35.1 (18.6)	17.1–99, *p* = 0.551	28.2 (3.57)	30 (4.80)	0–30.8, *p* = 0.967
***Mental Component Summary*** **(*MCS*)**	7.44 (3.88)	6.75 (4.10)	0–13.4, *p* = 0.588	24.1 (16.5)	21.8 (13.8)	0–50.1, *p* = 0.446	15.5 (9.73)	15.6 (8.45)	0–29.9, *p* = 0.517
***Physical Functioning*** **(*PF*)**	18.5 (5.70)	19.7 (5.10)	10–27.3, *p* = 0.494	15.3 (11.2)	17.5 (11.9)	0–40, *p* = 0.742	16 (8.68)	14.6 (9.33)	10–19.9, *p* = 0.719
***Role Physical*** **(*RP*)**	9.59 (2.33)	8.44 (2.15)	6.1–13.2, *p* = 0.395	10.4 (1.07)	10.5 (1.19)	9.2–12.3, *p* = 0.068	9.97 (10.5)	9.47 (5.95)	0–12, *p* = 0.409
***Bodily Pain*** **(*BP*)**	73.8 (4.20)	72.90 (4.09)	67.3–80, *p* = 0.143	13.5 (4.03)	11.6 (4.45)	6–20.5, *p* = 0.066	7.84 (3.97)	7 (4.15)	0–10.9, *p* = 0.847
***General Health*** **(*GH*)**	27.9 (4.69)	25.6 (4.38)	20.2–34, *p* = 0.419	73.9 (4.94)	71.6 (4.96)	63.8–80, *p* = 0.369	6.25 (2.69)	4.19 (3.43)	5.3–30, *p* = 0.010
***Vitality*** **(*VT*)**	42.7 (5.89)	42.1 (5.32)	34–52.5, *p* = 0.251	12 (6.46)	13.6 (7.58)	2–25, *p* = 0.578	14.6 (16.8)	18 (8.67)	10–16.3, *p* = 0.078
***Social Functioning*** **(*SF*)**	31.7 (3.51)	31.1 (3.37)	27.1–37, *p* = 0.471	18.8 (6.44)	17.9 (6.06)	8.1–30, *p* = 0.486	7.13 (4.24)	7.84 (4.40)	6.7–30, *p* = 0.508
***Role Emotional*** **(*RE*)**	77.7 (9.26)	77.3 (9.07)	60.4–90.2; *p* = 0.871	26.3 (12.6)	22.7 (13.2)	3.4–45, *p* = 0.269	25.9 (15)	24.8 (13.8)	1.2–50, *p* = 0.776
***Mental Health*** **(*MH*)**	62.8 (8.15)	63.8 (8.75)	51.3–78, *p* = 0.814	43.1 (24.5)	41.3 (26.1)	1–44, *p* = 0.772	6.88 (4.48)	6.41 (4.60)	2–14.3, *p* = 0.681
**CBI, mean (SD)**	20.51 (16.31)	23.60 (13.50)	17.99–25.45, *p* = 0.652	16.35 (17.26)	13.28 (14.11)	10.51–21.9, *p* = 0.486	24.22 (13,64)	24.89 (12.61)	11.2–25.3; *p* = 0.008
**ZBI, mean (SD)**	23.87 (15.86)	28.72 (16.08)	21.71–30.86, *p* = 0.150	18.16 (17.98)	14.00 (16.73)	11.42–20.60, *p* = 0.591	25.94 (15.81)	26.89 (14.60)	10.4–27.1; *p* = 0.847
**CBS, mean (SD)**	47.33 (22.43)	37.89 (23.71)	36.39–49.45, *p* = 0.206	31.00 (29.21)	23.78 (23.71)	10.70–28.77, *p* = 0.991	46.57 (19.93)	38.31 (17.85)	36.0–51.0; *p* = 0.269

*Abbreviations:* SD = standard deviation; OA = outpatient clinic adaptation (OA); HA = home adaptation; Cl = Class; SF-36 = Short Form Health Survey-36; CBI = Caregiver Burden Inventory; ZBI = Zarit Burden interview; CBS = Caregiving Burden Scale. In bold, find the items defined as significant.

**Table 4:** Quality of life and caregiver burden results within groups.

	Home Adaptation (HA)			Outpatient Clinic Adaptation (OA)		
Outcomes	Baseline	Follow-Up (2 Months Later)	95% Cl, *p*-Value	Baseline	Follow-Up (2 Months Later)	95% Cl, *p*-Value
**SF-36, mean (SD)**	26.78 (7.92)	52.21 (32.04)	**18.69–42.15, *p* = 0.000**	25.19 (9.71)	38.69 (21.63)	**4.11–22.88, *p* = 0.007**
**CBI, mean (SD)**	20.51 (16.31)	16.35 (17.26)	2.08–10.40, *p* = 0.183	23.60 (13.50)	13.28 (14.11)	**2.68–17.95, *p* = 0.010**
**ZBI, mean (SD)**	23.87 (15.86)	18.16 (17.98)	**0.070–11.42, *p* = 0.050**	28.72 (16.08)	14.00 (16.73)	**4.24–19.06, *p* = 0.003**
**CBS, mean (SD)**	47.33 (22.43)	31.00 (29.21)	**4.96–27.70, *p* = 0.008**	37.89 (23.71)	23.78 (23.71)	0.12–28.33, *p* = 0.052

*Abbreviations:* SD = standard deviation; OA = outpatient clinic adaptation; HA = home adaptation; SF-36 = Short Form Health Survey-36; CBI = Caregiver Burden Inventory; ZBI = Zarit Burden interview; CBS = Caregiving Burden Scale. In bold, find the items defined as significant.

## Data Availability

All data generated or analyzed during this study are included in this article and/or its supplementary material files. Further inquiries can be directed to the corresponding author.

## Supplementary Materials

The following are available online at https://www.mdpi.com/article/10.3390/jcm11113178/s1, Table S1: Educational Learning Test.

## Abbreviations

RCT = randomized controlled trial; ALS = amyotrophic lateral sclerosis; NIV = non-invasive ventilation; QoL = quality of life; ALSFRS-R = Amyotrophic Lateral Sclerosis Functional Rating Scale—Revized; BDS= Borg Dyspnoea Scale; ECAS = Edinburg Cognitive and Behavioural ALS Screen; ARF = acute respiratory failure; HA = home adaptation; OA = outpatient adaptation; MIP = maximum inspiratory pressure; MEP = maximum expiratory pressure; BMI = body mass index; RT = respiratory therapist; SpO2 = peripheral capillary oxygen saturation; ABG = arterial blood gas analysis; pH = measurement of acidity or alkalinity; PaCO2 = partial pressure of carbon dioxide; PaO2 = partial pressure of oxygen; HCO3 = bicarbonate; ETCO2 = end-tidal carbon dioxide; SBE = Subacute Bacterial Endocarditis, ESS = Epworth Sleepiness Scale; VAS = Visual Analogue Scale; SF-36 = 36-Item Short Form Survey; CBI = Caregiver Burden Inventory; CBS = Caregiver Burden Scale; ZBI = Zarit Burden Interview; SPSS = Statistical Package for Social Science; ANOVA= Analysis of Variance; aPCV = assisted pressure-controlled ventilation; AVAPS = average volume-assured pressure support.

## References

[1] Ricoy J., Rodríguez-Núñez N., Álvarez-Dobaño J.M., Toubes M.E., Riveiro V., Valdés L. (2019). Diaphragmatic dysfunction. Pulmonology.

[2] Andersen P.M., Abrahams S., Borasio G.D., de Carvalho M., Chio A., Van Damme P., Hardiman O., Kollewe K., Morrison K.E., EFNS Task Force on Diagnosis and Management of Amyotrophic Lateral Sclerosis (2012). EFNS guidelines on the Clinical Management of Amyotrophic Lateral Sclerosis (MALS)—Revised report of an EFNS task force. Eur. J. Neurol..

[3] Kleopa K.A., Sherman M., Neal B., Romano G.J., Heiman-Patterson T. (1999). Bipap improves survival and rate of pulmonary function decline in patients with ALS. J. Neurol. Sci..

[4] Piepers S., Berg J.V.D., Kalmijn S., Van Der Pol W., Wokke J.H.J., Lindeman E., Berg L.H.V.D. (2006). Effect of non-invasive ventilation on survival, quality of life, respiratory function and cognition: A review of the literature. Amyotroph. Lateral Scler..

[5] Vitacca M., Montini A., Lunetta C., Banfi P., Bertella E., De Mattia E., Lizio A., Volpato E., Lax A., Morini R. (2018). Impact of an early respiratory care programme with non-invasive ventilation adaptation in patients with amyotrophic lateral sclerosis. Eur. J. Neurol..

[6] Sheers N., Berlowitz D., Rautela L., Batchelder I., Hopkinson K., Howard M. (2014). Improved survival with an ambulatory model of non-invasive ventilation implementation in motor neuron disease. Amyotroph. Lateral Scler. Front. Degener..

[7] Luján M., Moreno A., Veigas C., Montón C., Pomares X., Domingo C. (2007). Non-invasive home mechanical ventilation: Effectiveness and efficiency of an outpatient initiation protocol compared with the standard in-hospital model. Respir. Med..

[8] Chatwin M., Nickol A.H., Morrell M.J., Polkey M.I., Simonds A.K. (2008). Randomised trial of inpatient versus outpatient initiation of home mechanical ventilation in patients with nocturnal hypoventilation. Respir. Med..

[9] Bertella E., Banfi P., Paneroni M., Grilli S., Bianchi L., Volpato E., Vitacca M. (2017). Early initiation of night-time NIV in an outpatient setting: A randomized non-inferiority study in ALS patients. Eur. J. Phys. Rehabil. Med..

[10] Kim C.H., Kim M.S. (2014). Ventilator use, respiratory problems, and caregiver well-being in Korean patients with amyotrophic lateral sclerosis receiving home-based care. J. Neurosci. Nurs..

[11] Dorst J., Ludolph A.C. (2019). Non-invasive ventilation in amyotrophic lateral sclerosis. Ther. Adv. Neurol. Disord..

[12] Pinto A., Almeida J.P., Pinto S., Pereira J., Oliveira A., de Carvalho M. (2010). Home telemonitoring of non-invasive ventilation decreases healthcare utilisation in a prospective controlled trial of patients with amyotrophic lateral sclerosis. J. Neurol. Neurosurg. Psychiatry.

[13] Heiman-Patterson T.D., Cudkowicz M.E., De Carvalho M., Genge A., Hardiman O., Jackson C.E., Lechtzin N., Mitsumoto H., Silani V., Andrews J.A. (2018). Understanding the use of NIV in ALS: Results of an international ALS specialist survey. Amyotroph. Lateral Scler. Front. Degener..

[14] Kimura F., Shinoda K., Fujiwara S., Fujimura C., Nakajima H., Furutama D., Sugino M., Hanafusa T. (2003). The changes of clinical characteristics in 100 Japanese amyotrophic lateral sclerosis patients between 1980 and 2000. Rinsho Shinkeigaku.

[15] Tan G.P., Soon L.H.Y., Ni B., Cheng H., Tan A.K.H., Kor A.C., Chan Y. (2019). The pattern of use and survival outcomes of a dedicated adult Home Ventilation and Respiratory Support Service in Singapore: A 7-year retrospective observational cohort study. J. Thorac. Dis..

[16] Chio A., Gauthier A., Calvo A., Ghiglione P., Mutani R. (2005). Caregiver burden and patients’ perception of being a burden in ALS. Neurology.

[17] Pagnini F., Rossi G., Lunetta C., Banfi P., Castelnuovo G., Corbo M., Molinari E. (2010). Burden, depression, and anxiety in caregivers of people with amyotrophic lateral sclerosis. Psychol. Health Med..

[18] Ludolph A., Drory V., Hardiman O., Nakano I., Ravits J., Robberecht W., Shefner J. (2015). A revision of the El Escorial criteria-2015. Amyotroph Lateral Scler Front. Degener.

[19] Abrahams S., Bak T. (2013). Edinburgh Cognitive and Behavioural ALS Screen-ECAS English Version 2013. https://era.ed.ac.uk/handle/1842/6592.

[20] Siciliano M., Trojano L., Trojsi F., Greco R., Santoro M., Basile G., Piscopo F., D’Iorio A., Patrone M., Femiano C. (2017). Edinburgh Cognitive and Behavioural ALS Screen (ECAS)-Italian version: Regression based norms and equivalent scores. Neurol. Sci..

[21] Cedarbaum J.M., Stambler N., Malta E., Fuller C., Hilt D., Thurmond B., Nakanishi A. (1999). The ALSFRS-R: A revised ALS functional rating scale that incorporates assessments of respiratory function. J. Neurol. Sci..

[22] Just N., Bautin N., Danel-Brunaud V., Debroucker V., Matran R., Perez T. (2010). The Borg dyspnoea score: A relevant clinical marker of inspiratory muscle weakness in amyotrophic lateral sclerosis. Eur. Respir. J..

[23] Johns M.W. (1991). A New Method for Measuring Daytime Sleepiness: The Epworth Sleepiness Scale. Sleep.

[24] Pellegrino G.M., Papa G.F.S., Centanni S., Corbo M., Kvarnberg D., Tobin M.J., Laghi F. (2021). Measuring vital capacity in amyotrophic lateral sclerosis: Effects of interfaces and reproducibility. Respir. Med..

[25] Quanjer P.H., Tammeling G.J., Cotes J.E., Pedersen O.F., Peslin R., Yernault J.C. (1993). Lung volumes and forced ventilatory flows: Report of Working Party “Standardization of Lung Function Test”. Eur. Respir. J..

[26] Chaudri M., Liu C., Hubbard R., Jefferson D., Kinnear W. (2002). Relationship between supramaximal flow during cough and mortality in motor neurone disease. Eur. Respir. J..

[27] Lalmolda C., Prados H., Mateu G., Noray M., Pomares X., Lujánab M. (2019). Titration of Mechanical Insufflation–Exsufflation Optimal Pressure Combinations in Neuromuscular Diseases by Flow/Pressure Waveform Analysis. Arch. De Bronconeumol..

[28] American Academy of Sleep Medicine Task Force (1999). Sleep-related breathing disorders in adults: Recommendations for syndrome definition and measurement techniques in clinical research. Sleep.

[29] Berry R.B., Budhiraja R., Gottlieb D.J., Gozal D., Iber C., Kapur V.K., Marcus C.L., Mehra R., Parthasarathy S., Quan S.F. (2012). Rules for Scoring Respiratory Events in Sleep: Update of the 2007 AASM Manual for the Scoring of Sleep and Associated Events. Deliberations of the Sleep Apnea Definitions Task Force of the American Academy of Sleep Medicine. J. Clin. Sleep Med..

[30] Brazier J.E., Harper R., Jones N.M., O’Cathain A., Thomas K.J., Usherwood T., Westlake L. (1992). Validating the SF-36 health survey questionnaire: New outcome measure for primary care. BMJ.

[31] Novak M., Guest C. (1989). Application of a multidimensional caregiver burden inventory. Gerontologist.

[32] Gupta R. (1999). The Revised Caregiver Burden Scale: A Preliminary Evaluation. Res. Soc. Work Pract..

[33] Chattat R., Cortesi V., Izzicupo F., Del Re M.L., Sgarbi C., Fabbo A., Bergonzini E. (2011). The Italian version of the Zarit Burden Interview: A validation study. Int. Psychogeriatr..

[34] Gonzalez-Bermejo J., Perrin C., Janssens J.-P., Pepin J., Mroue G., Léger P., Langevin B., Rouault S., Rabec C., Rodenstein D. (2012). Proposal for a systematic analysis of polygraphy or polysomnography for identifying and scoring abnormal events occurring during non-invasive ventilation. Thorax.

[35] Morelot-Panzini C., Bruneteau G., Gonzalez-Bermejo J. (2019). NIV in amyotrophic lateral sclerosis: The ‘when’ and ‘how’ of the matter. Respirology.

[36] Bach J.R. (2002). Amyotrophic lateral sclerosis: Prolongation of life by noninvasive respiratory AIDS. Chest.

[37] Kim S.-M., Park K.S., Nam H., Ahn S.-W., Kim S., Sung J.-J., Lee K.-W. (2011). Capnography for Assessing Nocturnal Hypoventilation and Predicting Compliance with Subsequent Noninvasive Ventilation in Patients with ALS. PLoS ONE.

[38] Erdfelder E., Faul F., Buchner A. (1996). GPOWER: A general power analysis program. Behav. Res. Methods Instrum. Comput..

[39] Hazenberg A., Kerstjens H., Prins S., Vermeulen K., Wijkstra P. (2014). Initiation of home mechanical ventilation at home: A randomised controlled trial of efficacy, feasibility and costs. Respir. Med..

[40] Duiverman M.L., Vonk J.M., Bladder G., Van Melle J.P., Nieuwenhuis J., Hazenberg A., Kerstjens H., van Boven J., Wijkstra P.J. (2020). Home initiation of chronic non-invasive ventilation in COPD patients with chronic hypercapnic respiratory failure: A randomised controlled trial. Thorax.

[41] Van den Biggelaar R.J., Hazenberg A., Cobben N.A., Gaytant M.A., Vermeulen K.M., Wijkstra P.J. (2020). A Randomized Trial of Initiation of Chronic Noninvasive Mechanical Ventilation at Home vs In-Hospital in Patients With Neuromuscular Disease and Thoracic Cage Disorder: The Dutch Homerun Trial. Chest.

[42] Markovic N., Povitz M., Smith J., Leasa D., Shoesmith C., Gofton T.E. (2018). Patterns of Non-Invasive Ventilation in Amyotrophic Lateral Sclerosis. Can. J. Neurol. Sci. J. Can. des Sci. Neurol..

[43] Manera U., Torrieri M.C., Moglia C., Canosa A., Vasta R., Ribolla F., Palumbo F., Solero L., Mora G., Mattei A. (2021). Arterial blood gas analysis: Base excess and carbonate are predictive of noninvasive ventilation adaptation and survival in amyotrophic lateral sclerosis. Amyotroph. Lateral Scler. Front. Degener..

[44] Sahni A.S., Wolfe L. (2018). Respiratory Care in Neuromuscular Diseases. Respir. Care.

[45] Hazenberg A., Kerstjens H.A., Prins S.C., Vermeulen K.M., Wijkstra P.J. (2016). Is chronic ventilatory support really effective in patients with amyotrophic lateral sclerosis?. J. Neurol..

[46] Ando H., Chakrabarti B., Angus R.M., Cousins R., Thornton E.W., Young C. (2014). Experience of long-term use of non-invasive ventilation in motor neuron disease: An interpretative phenomenological analysis. BMJ Support. Palliat. Care.

[47] Ando H., Williams C., Angus R.M., Thornton E.W., Chakrabarti B., Cousins R., Piggin L.H., Young C. (2015). Why don’t they accept non-invasive ventilation?: Insight into the interpersonal perspectives of patients with motor neurone disease. Br. J. Health Psychol..

[48] Cousins R., Ando H., Thornton E., Chakrabarti B., Angus R., Young C. (2013). Determinants of accepting non-invasive ventilation treatment in motor neurone disease: A quantitative analysis at point of need. Health Psychol. Behav. Med..

[49] MacIntyre E.J., Asadi L., Mckim D.A., Bagshaw S.M. (2016). Clinical Outcomes Associated with Home Mechanical Ventilation: A Systematic Review. Can. Respir. J..

[50] Bourke S.C., Tomlinson M., Williams T.L., Bullok R.E., Shaw P.J., Gibson G.J. (2006). Effects of non-invasive ventilation on survival and quality of life in patients with amyotrophic lateral sclerosis: A randomized controlled trial. Lancet Neurol..

[51] Meng L., Bian A., Jordan S., Wolff A., Shefner J.M., Andrews J. (2018). Profile of medical care costs in patients with amyotrophic lateral sclerosis in the Medicare programme and under commercial insurance. Amyotroph. Lateral Scler. Front. Degener..

